# Effects of Washing Conditions on PAH Removal Effectiveness in Firefighter Protective Clothing Materials

**DOI:** 10.3390/ma18174073

**Published:** 2025-08-30

**Authors:** Sylwia Maria Krzemińska, Małgorzata Szewczyńska, Pamela Miśkiewicz, Witold Sygocki

**Affiliations:** 1 Department of Personal Protective Equipment, Central Institute for Labour Protection-National Research Institute, Czerniakowska 16, 00-701 Warsaw, Poland; 2 Department of Chemical, Biological and Aerosol Hazards, Central Institute for Labour Protection-National Research Institute, Czerniakowska 16, Warsaw 00-701, Poland; 3 Institute of Architecture of Textiles, Faculty of Material Technologies and Textile Design, Lodz University of Technology, Żeromskiego 116, Lodz 90-924, Poland; pamela.miskiewicz@p.lodz.pl; 4 Centre for Evaluation and Scientific Communication, Central Institute for Labour Protection-National Research Institute, Czerniakowska 16, Warsaw 00-701, Poland; wisyg@ciop.pl

**Keywords:** protective clothing, firefighting, polycyclic aromatic hydrocarbons (PAHs), contamination, washing effectiveness, washing

## Abstract

This study analyzes the effects of washing conditions on polycyclic aromatic hydrocarbon (PAH) content in firefighter protective clothing. The analysis involved specially prepared textile packages made of materials used in such clothing: an outer shell, a moisture barrier membrane, and a thermal insulation lining. Package samples were subjected to simulated exposure to a selected group of PAH compounds. Ultra-high-performance liquid chromatography with fluorescence detection (UHPLC/FL) was applied to determine PAH content. The study showed that washing conditions (water temperature and the number of rinses) influenced the effectiveness of removal of chemical contaminants. The most favorable results were obtained for the washing process conducted at 60 °C with three rinse cycles, which resulted in the lowest concentration of total PAHs in the two examined types of textile packages (0.40 µg·g^−1^ and 0.60 µg·g^−1^ in the outer shell, 3.9 µg·g^−1^ and 6.2 µg·g^−1^ in the membrane, and 0.40 µg·g^−1^ and 0.41 µg·g^−1^ in the thermal lining of packages A and B, respectively). The higher washing temperature (60 °C) had a more favorable effect on average washing effectiveness as compared with the lower temperature (40 °C) in both the two- and three-rinse variants. The average washing effectiveness also varied according to the type of material and amounted to 70% and 54% for textile package types A and B, respectively.

## 1. Introduction

In recent years, there has been increased interest in the health problems of firefighters resulting from exposure to harmful chemicals. Research shows that such substances are adsorbed on the clothing and personal protective equipment (PPE) used by firefighters. Those contaminants typically include polycyclic aromatic hydrocarbons (PAHs), volatile organic compounds, and particulate matter, as well as per- and polyfluoroalkyl substances [1,2,3]. Studies have demonstrated a relationship between the conditions in which firefighters perform rescue and firefighting operations, the degree of exposure to harmful chemicals, and the risk of chronic diseases, including cancer. A meta-analysis by Casjens et al. [4] found an increased incidence of skin melanoma and prostate cancer among firefighters from different geographic areas. Furthermore, Pukkala et al. [5], who conducted a study involving approx. 16,500 Nordic firefighters, observed a correlation between melanoma and exposure to benzene and PAHs (e.g., acenaphthene, benzo(a)anthracene, benzo(a)pyrene, and benzo(e)pyrene). Since 2022, the International Agency for Research on Cancer has classified chemicals to which firefighting professionals are exposed as “carcinogenic” (group 1) [6]. An analysis by Stec et al. [6] concluded that exposure to chemicals released during fires is a significant risk factor for cancer and other diseases among firefighters. A review paper by Orysiak et al. [7] also found a higher incidence of other types of diseases among firefighters, such as cardiovascular and respiratory conditions and cancers due to inflammation, mainly resulting from exposure to fires. The inhalation of smoke particles by firefighters can trigger an inflammatory response in the respiratory system, possibly leading to infections (such as bronchitis or pneumonia), and this in turn may contribute to the development of lung cancer [7].

Given the layered design of protective clothing for firefighters (consisting of an outer shell, waterproof membrane, and thermal insulation), chemical contaminants penetrate the inner layers from the outer ones [8,9]. Each layer is characterized by a different set of properties. The outer shell provides protection against flames, heat radiation, and liquid splashes. The middle layer (moisture barrier) serves as a barrier against liquid penetration and assists the transport of sweat to the outer surface of the garment. The inner layer constitutes thermal insulation, providing comfort by minimizing thermal stress and removing moisture from the skin [10]. Most of the materials used in firefighter protective clothing are made of porous fabrics permeable to chemicals [11]. Chemical molecules can also penetrate the constituent fibers of the materials and remain there for a long time [8,12,13]. In addition, the highly lipophilic nature of PAHs enhances their absorption into the human body through different pathways, such as inhalation and skin contact [14,15].

Recently, much attention has been paid to per- and polyfluoroalkyl substances (PFASs), which pose a health risk to firefighters, as well as to other professional groups and the natural environment. Due to their carbon–fluorine bonds, these compounds are characterized by high thermal and chemical stability, hydrophobicity, and oil resistance [16,17,18]. Preliminary studies by Peaslee et al. [19] indicate that firefighter protective clothing materials release significant amounts of fluorinated compounds throughout their lifecycle. The level of firefighters’ exposure to PAHs and PFASs has also been studied by biomonitoring changes in their concentrations in the blood and urine after drills and exposure to flames. Řiháčková et al. [20] found that overall PAH concentration levels in firefighters’ urine increased significantly after exercises involving burning wooden pallets, but did not reach the level of genotoxicity. Therefore, it is so important to properly clean protective clothing after use to reduce the level of chemical contamination [21]. Such substances are typically removed from protective clothing by laundering after use. Stull [11], who studied the effectiveness of industrial laundering of firefighter protective clothing, found that washing significantly reduced the amount of contamination, depending on the type of chemical. In turn, Banks et al. [22] reported significant reductions in PAH concentrations after washing or sanitizing for 3 of 16 pieces of tested clothing. However, the impact of laundering process conditions on washing effectiveness for chemicals accumulated in clothing materials has not yet been fully recognized. A large role is played by secondary contamination occurring during the washing process.

In our previous work [21] we determined PAH content in firefighter protective clothing, particularly in the outer shell, moisture barrier membrane, and thermal barrier, following fire suppression operations and live-fire enclosure simulations. Samples were taken from clothing pre-fire, post-fire, and post-washing. The material samples were subjected to one washing cycle using a constant number of rinse cycles. Moreover, the study investigated differences in PAH content between the various locations of clothing (the elbow region in sleeves and the knee region in legs).

The aim of this study was to analyze the effects of washing conditions, differing in terms of temperature (40 °C or 60 °C) and the number of rinse cycles (two or three), on PAH content determined in firefighter protective clothing materials. The study used specially prepared textile packages made of such materials: the outer shell, the moisture barrier membrane, and the thermal barrier. The packages were subjected to simulated contamination with PAHs by depositing onto them a specified volume of a PAH solution. This pretreatment was intended to minimize variation in contamination levels found in real life following rescue operations and to more precisely determine the effects of washing process conditions on the effectiveness of removing harmful PAH compounds.

## 2. Materials and Methods

### 2.1. Materials

The study involved materials from two types of firefighter protective clothing (Subor, Staszów, Poland). Both of them consisted of three material layers: the outer shell, the middle moisture barrier membrane, and the inner thermal insulation layer. The two types of clothing, designated as A and B, differed in terms of the composition of the materials used (Table 1).

**Figure 1:**
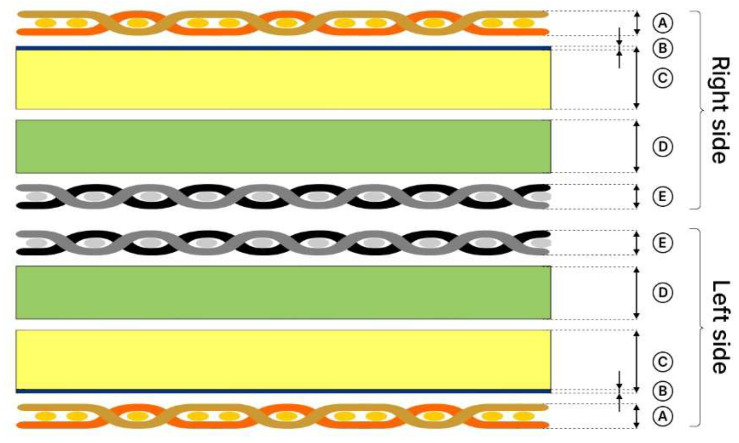
Cross-section of the packages prepared for testing (A—outer shell; B—moisture barrier membrane; C—fleece membrane; D—thermal insulation felt; E—thermal insulation lining). Different colors represent different layers.

**Figure 2:**
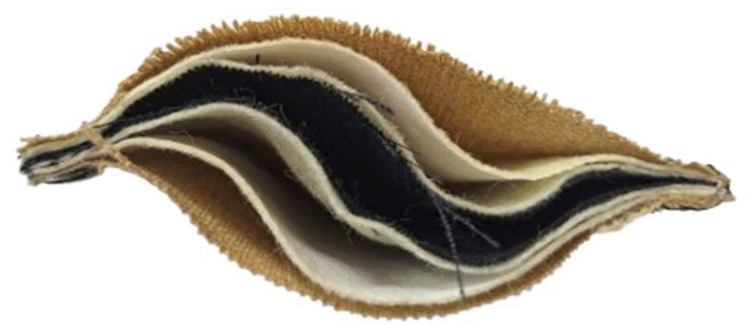
A photograph of a cross-section of a prepared material package.

The tested samples were packages consisting of two firefighter protective clothing material types (A or B). Each type contained three layers: an outer shell, a middle moisture membrane, and an inner thermal insulation lining (Figure 1). In the study, the two package types were sewn together so that their thermal insulation layers were adjacent, with the outer shell on the outside. The materials were arranged in the same order as in protective clothing (Figure 2).

It was assumed that the top of these assemblies imitated the front of firefighter protective clothing, while the bottom imitated the back of clothing.

The edges of the material packages were sewn together to prevent delamination during testing. Twelve 80 × 80 mm or 95 × 95 mm packages were prepared from each material type (A, B). Three packages were used for testing under each washing process condition. Additionally, tests on new samples were conducted using additional material packages, three packages from each type of material (A, B).

### 2.2. Procedure of Simulated PAH Exposure

**Figure 3:**
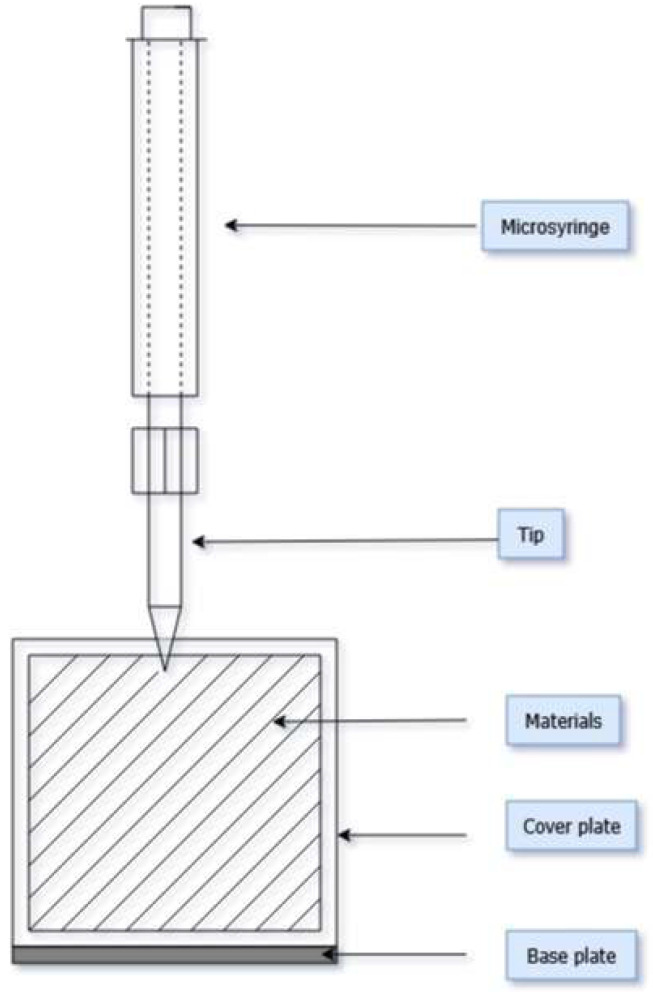
Scheme of simulated exposure to PAH solution of packages comprising firefighter protective clothing materials.

**Figure 4:**
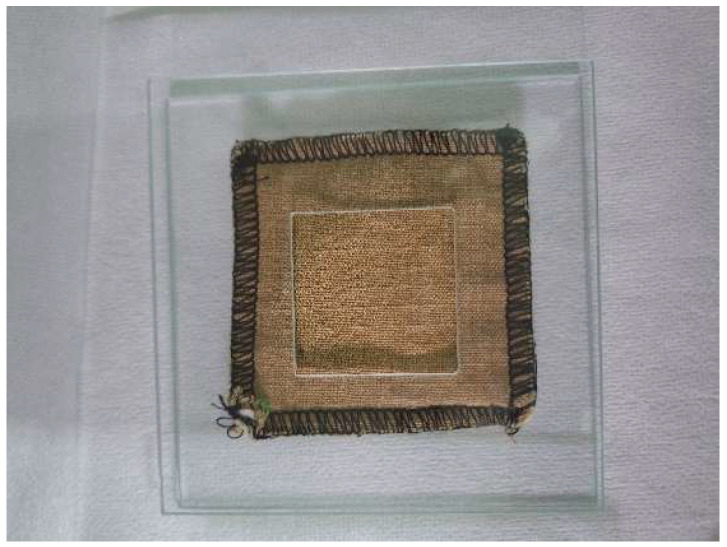
A photograph of a material package placed between glass plates (material type B).

New material packages were subjected to simulated exposure to a solution containing PAH compounds. Each package was placed between a baseplate and a cover plate (Figure 3). Filter paper was placed on the baseplate, and the package was placed on the paper so that its top side faced upwards. The package was pressed against the baseplate with a cover plate that had a square opening in its center (Figure 4). Then, a fixed volume (250 µL) of a PAH working standard solution was applied to the center of the package with a microsyringe. The time of simulated exposure to PAH was 20 min. Subsequently, the cover plate was removed, and the package was prepared for the washing and drying process, which was performed according to the recommendations provided by the garment manufacturer in the user’s instructions. After completing the process, the package was subjected to PAH content analysis. Samples of materials (the outer shell, membrane, and thermal insulation), ranging in size from 40 × 40 cm to 70 × 70 mm, were cut from the package. For comparison purposes, packages not exposed to the chemical solution were also subjected to the washing process.

EPA 610 Polynuclear Aromatic Hydrocarbons Mix 100–2000 µg-mL^−1^ MEOH:CH2Cl2(1:1) (Sigma-Aldrch Supelco, Bellefonte, PA, USA) was used as a certified set of PAH standards (Table 2) to treat the material packages (Table 2). These standards were also used to calibrate the analytical equipment.

### 2.3. Washing Procedure

**Figure 5:**
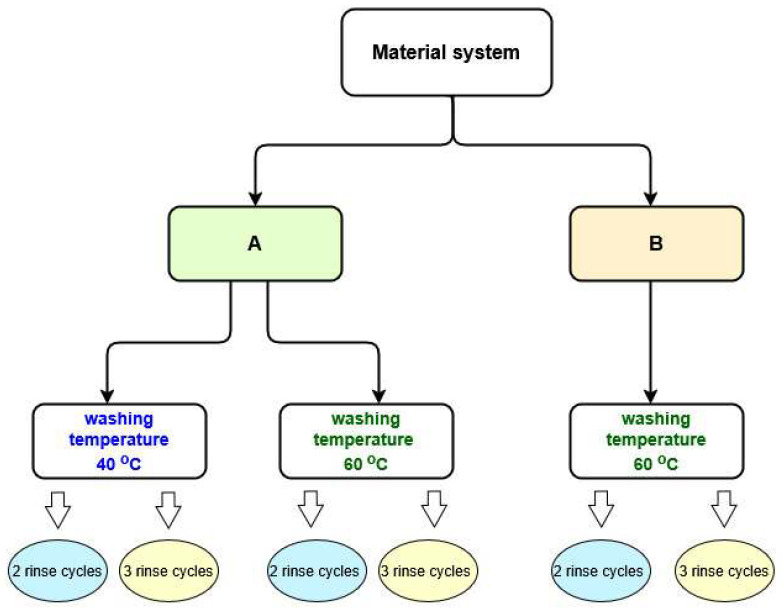
Variants of the washing conditions applied.

In order to assess the impact of washing conditions on the effectiveness of removal of the selected group of harmful chemicals (PAHs) from firefighter protective clothing, the material packages were washed at different temperatures and using different numbers of rinses. The standard recommendations of the protective clothing manufacturer were expanded by introducing additional variants at different washing temperatures and with different numbers of rinses (Figure 5). The washing process was carried out using the following 4 variants:

1. Temperature 60 °C, 2 rinse cycles—as recommended by the clothing manufacturer.
2. Temperature 40 °C, 2 rinse cycles.
3. Temperature 60 °C, 3 rinse cycles.
4. Temperature 40 °C, 3 rinse cycles.

The study involved a specialized washing procedure with a horizontal drum, designed for laundering firefighter protective clothing (PWT 6089, Miele, Gütersloh, Germany). The washing procedure consisted of a 50 min washing cycle (with 2 rinses) or a 60 min washing cycle (with 3 rinses) followed by tumble drying at 40 °C for 120 min. Before each washing procedure, an empty run was carried out with water only, prior to a run with ballast material (pieces of fabric filling the washing machine drum).

The applied washing agents were detergents dedicated specifically to the washing used. Special laundry detergents were acquired from the same company (Derval “Greenpol,” Institute of Environmental Design Sp. z o.o., Zielona Gora, Poland). They consisted of a basic detergent without bleach and a detergent containing fortifying enzymes.

### 2.4. Cross-Contamination

To minimize cross-contamination,

- Each set of three packages was washed separately in the washing process under specified conditions (temperature, number of rinse cycles);
- Each set of packages was washed in the same machine;
- The machine was cleaned between each washing cycle by running a standard washing cycle;
- Each day, an empty run was made with water and detergents before washing began.

### 2.5. Chemicals

The following reagents were used in the study: acetonitrile, dichloromethane (J.T. Baker, USA), MilliQ ultrapure water (Millipore, Darmstadt, Germany), and a set of certified PAH standards—EPA 610 Polynuclear Aromatic Hydrocarbons Mix 100–2000 μg·mL^−1^ MEOH:CH2Cl2(1:1) (Sigma-Aldich Supelco, Bellefonte, PA, USA).

### 2.6. Methods

Analytical procedure

PAHs were determined by ultra-high-performance liquid chromatography with fluorescence detection (UHPLC/FL) using an EliteLaChrom Ultra liquid chromatograph coupled to a fluorescence detector (Merck-Hitachi, Darmstadt, Germany) and a Pinnacle II PAH analytical column (Restek, Bellefonte, PA, USA) with a length of 150 mm and an internal diameter of 3.2 mm. The detailed conditions of analysis were described in a previous publication [21].

Preparation of calibration curves, blank samples, and loss of PAHs

Calibration curves were made in the concentration range of 0.0025–1.0000 μg·mL^−1^. To prepare the curves, six 0.04 × 0.04 m samples were cut from each of the three material layers (outer shell, middle membrane, inner thermal insulation) from a new piece of protective clothing for firefighters.

Samples were placed in separate 25 mL Erlenmeyer flasks, and 100 μL of PAH working standard solution was transferred onto each sample. A procedure described in detail in a previous publication [21] was used to make calibration curves, test blanks, and recover PAHs.

### 2.7. Sample Preparation

Samples taken from the material packages after the washing process were prepared for PAH analysis and analyzed in the same way as the samples used to prepare the calibration curves, except that they were not pretreated with a PAH solution.

### 2.8. Statistical Analyses

Statistical analysis of the results was conducted to assess the significance of the effects of the washing temperature and number of rinse cycles on the total PAH concentration and the effectiveness of the washing process.

The following statistical methods were used:

- *t*-test—To test the significance of differences between two groups when the condition of normality of distribution and the condition of homogeneity of variance were met;
- Cochran–Cox test—To test the significance of differences between two groups when the condition of normality of distribution was met but the condition of homogeneity of variance was not;
- Analysis of variance (ANOVA)—To test the significance of differences between at least three groups when the conditions of normality of distribution and homogeneity of variance were met;
- Welch’s *F* test—To test the significance of differences between at least three groups when the condition of normality of distribution was met but the condition of homogeneity of variance was not.

The Tukey test was used as a post hoc test to check the significance of differences between pairs of groups. The normality of distribution was evaluated with the Shapiro–Wilk test, and the condition of homogeneity of variance with the Levene test.

The significance threshold was set at α = 0.05. Results were deemed statistically significant for probability values of *p* ≤ 0.05. The resultant PAH concentration data were analyzed using Statistica version 10.0 software (Statsoft, Kraków, Poland).

## 3. Results and Discussion

### 3.1. Effect of Washing Temperature

#### 3.1.1. Effect of Material Washing Temperature on Individual PAH Concentrations

**Figure 6:**
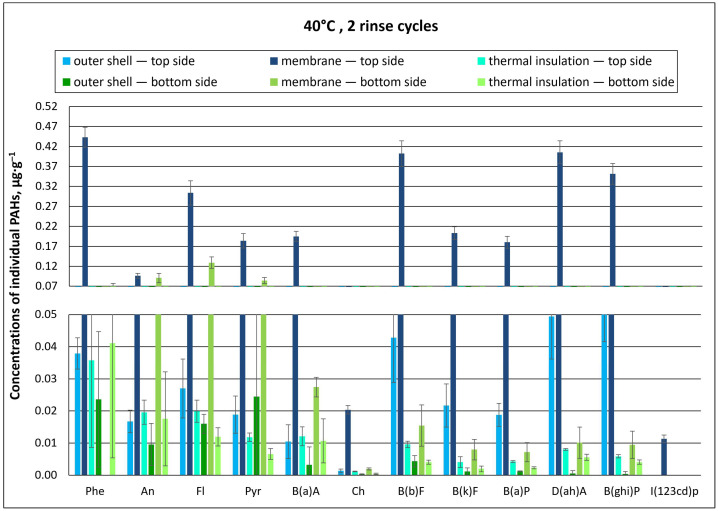
Concentrations of individual PAHs for type A material packages, subjected to chemical exposure followed by washing (40 °C, 2 rinse cycles; error bars represent standard deviations).

**Figure 7:**
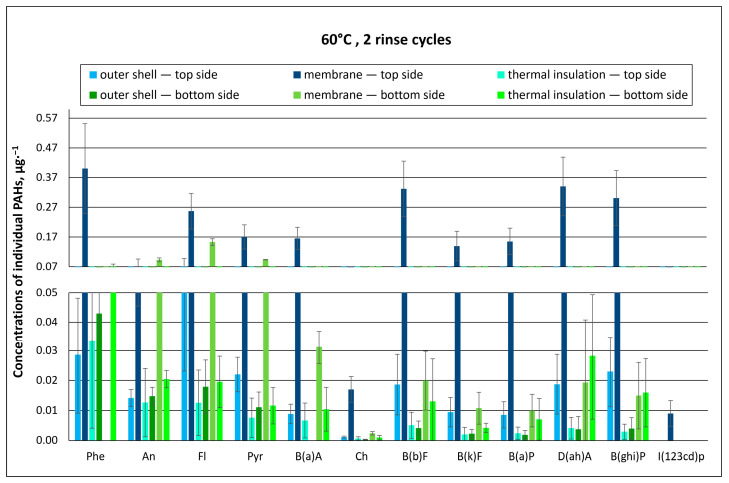
Concentrations of individual PAHs for type A material packages, subjected to chemical exposure followed by washing (60 °C, 2 rinse cycles; error bars represent standard deviations).

The concentrations of individual PAHs found for material type A are summarized in Figure 6 and Figure 7, as well as in the Supplementary Materials (Tables S1 and S2).

Regardless of the temperature of the washing process (40 °C or 60 °C), PAH concentrations found for the top side of type A packages were many times higher than those found on their bottom side. This indicates that the PAH solution applied onto the packages accumulated in the outer materials of the top of the package and further penetrated the inner layers. The highest concentrations of individual PAHs were determined in the membrane layer after washing at both 40 °C and 60 °C with two rinse cycles (Figure 6 and Figure 7). The membrane, probably due to its structure, adsorbed and retained PAHs, which are not easily degradable due to their properties. Therefore, it was difficult to remove them at both temperatures.

PAH concentrations in the membrane of the top side of packages washed at 40 °C were as follows: phenanthrene (Phe)—0.44 µg∙g^−1^; dibenzo(ah)anthracene D(ah)A—0.41 µg∙g^−1^; and benzo(b)fluoranthene B(b)F—0.40 µg∙g^−1^. In contrast, PAH concentrations for the bottom side of the package were: fluoranthene (Fl)—0.13 µg∙g^−1^; anthracene (An)—0.091 µg∙g^−1^; and pyrene (Pyr)—0.084 µg∙g^−1^ (Figure 6). The lowest concentrations were recorded in the thermal barrier layer: chrysene (Ch) at 0.001 µg∙g^−1^ and benzo(k)fluoranthene B(k)F and benzo(a)pyrene B(a)P at 0.004 µg∙g^−1^ (Figure 6). It should be noted here that dibenzo(a,h)anthracene had the highest relative carcinogenicity index (RCI) of 5. In contrast, the RCI for B(a)P, which was considered an indicator compound for the PAH group, was 1. For the other analyzed PAHs, RCI values were much lower and ranged between 0.001 and 0.1 [24,25].

In the washing process conducted at the higher temperature (60 °C), the concentrations of individual PAHs in the packages were also the highest in the membrane (Figure 7). On the bottom side of packages washed at 60 °C, individual PAH concentrations were several times lower as compared to the top side, as was the case for packages washed at 40 °C. The highest concentration values were recorded in membrane samples: 0.15 µg∙g^−1^ for fluoranthene (Fl), 0.094 µg∙g^−1^ for pyrene (Pyr), and 0.093 µg∙g^−1^ for anthracene (An) (Figure 7).

In previous studies [21], high concentrations of PAHs, such as benzo(ghi)perylene (B(ghi)P), indeno(1,2,3-cd)pyrene (I(123cd)P), and benzo(b)fluoranthene (B(b)F), were found in samples of firefighter protective clothing used during rescue operations and subjected to a washing process in a laboratory laundry at 60 °C. The concentration of B(ghi)P reached 8.1 µg∙g^−1^ in the outer shell, 20.7 µg∙g^−1^ in the moisture membrane, and 0.2 µg∙g^−1^ in the thermal insulation. In contrast, the concentration of I(123cd)P reached 9.1 µg∙g^−1^ in the outer shell, 11.5 µg∙g^−1^ in the moisture membrane, and 0.3 µg∙g^−1^ in the thermal insulation [21]. These concentrations were significantly higher than those in the studies discussed above for package samples. However, there was an apparent trend in which the highest content of PAH contaminants was determined in the membrane layer, regardless of whether it was used in protective clothing or in specially prepared material packages. This indicates that the membrane, due to its structure, accumulates the highest amounts of chemical contaminants.

Research in a similar area was conducted by Forester et al. [26], who determined the effectiveness of removing PAHs from structural firefighter gear at different washing temperatures (40 °C, 52 °C, and 60 °C). They found that a washing temperature of 40 °C was sufficient to remove volatile and semi-volatile chemicals (chlorinated phenols) at a washing effectiveness of over 85%. However, washing at 40 °C was insufficient for PAHs, for which the washing effectiveness ranged between 20% and 50%. Forester et al. [26] determined the content of one of the PAHs, phenanthrene, in samples at 186, 134, and 63 ng·cm^−2^, respectively, for washing temperatures of 40 °C, 52 °C, and 60 °C. It was observed that the washing temperature had a significant effect on the removal of chemical contaminants.

The determined concentrations of the tested PAHs did not exceed the limits specified in the AfPS GS 2019:01 PAK document issued by the German Committee for Product Safety regarding PAH content in products [27]. According to the standard, category 2b products, i.e., those which come into contact with the skin for over 30 s (or less if skin exposure is recurrent), the content of a single PAH should be below 0.5 µg·g^−1^. The same requirements were reiterated in a document adopted by the Oeko-Tex Organization [28].

#### 3.1.2. Effect of Washing Temperature on Total PAH Concentration

**Figure 8:**
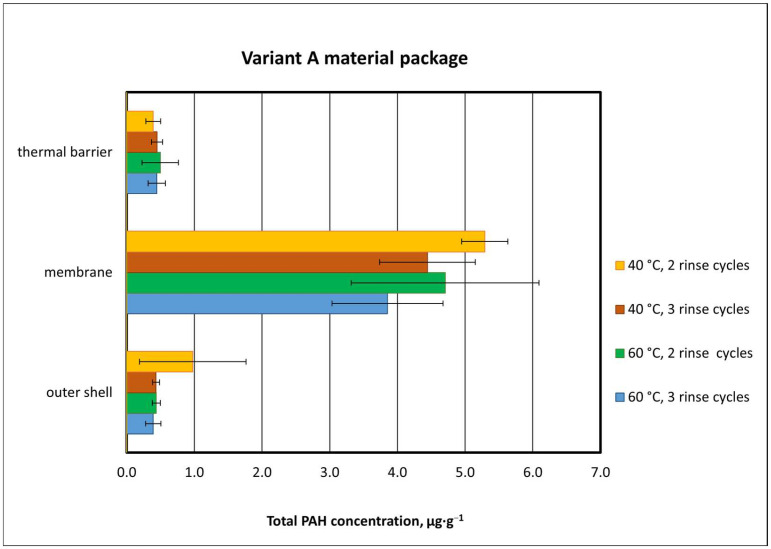
Total PAH concentrations in type A material packages after exposure to chemicals and washing (results and standard deviations).

Analyzing the effects of washing temperature on the degree of removal of chemical contaminants from type A packages, it was found that the washing process at the lower temperature of 40 °C led to higher total PAH concentrations in the materials, especially in the outer shell and membrane (Figure 8). In the washing process with two rinse cycles, total PAH concentrations in the outer shell were 0.98 µg·g^−1^ at 40 °C and 0.44 µg·g^−1^ at 60 °C.

A washing temperature of 60 °C reduced the concentration of total PAHs determined in the outer shell by about 50%, but the difference was not statistically significant (*p* = 0.359). The total PAH concentration determined in the membrane layer after washing at 60 °C was 5.29 µg·g^−1^, approx. 12% lower than that after washing at 40 °C (Figure 8). A different situation was observed for the thermal insulation layer, where a higher washing temperature did not lead to higher effectiveness. The total PAH concentration determined in the thermal insulation after washing was 0.39 µg·g^−1^ at 40 °C and 0.44 µg·g^−1^ at 40 °C. The difference was approx. 14%.

When three rinse cycles were used, it was noted that the washing temperature affected total PAH concentrations only for one layer, i.e., the membrane (Figure 8). Similarly, as for two rinse cycles, lower total PAH concentrations were found for a washing temperature of 60 °C. The difference in total PAH concentrations in membranes between washing at 40 °C and 60 °C was 0.59 µg-g^−1^, but still it failed to reach statistical significance (*p* = 0.602). For the other material layers (outer shell, thermal barrier), the application of a third rinse cycle did not change total PAH concentrations.

A study of the total concentration of 13 PAH compounds in firefighter underwear materials was conducted by Engelsman and collaborators [29], who assessed contamination and laundering effectiveness for underwear used by firefighters during a specific scenario of activities during a simulated fire. Similarly to our study, samples were cut from the cuffs of socks and the front of underwear and tops after use and after washing. The laundering process was carried out in a household-type washing machine at 60 °C using four different household laundry detergents. The obtained PAH concentrations were 2.6, 1.2, and 0.5 µg·g^−1^ for samples from socks, underwear, and tops, respectively [29]. These concentrations were similar to those obtained in our study of firefighter protective clothing materials. However, it should be noted that the difference in total PAH concentration in protective clothing materials was greater (0.5·5 µg·g^−1^). The authors concluded that this was due to the position of the material in the layered design (outer shell, membrane, thermal insulation).

### 3.2. Effect of the Number of Rinse Cycles

#### 3.2.1. Effect of the Number of Rinse Cycles on the Concentration of Individual PAHs

**Figure 9:**
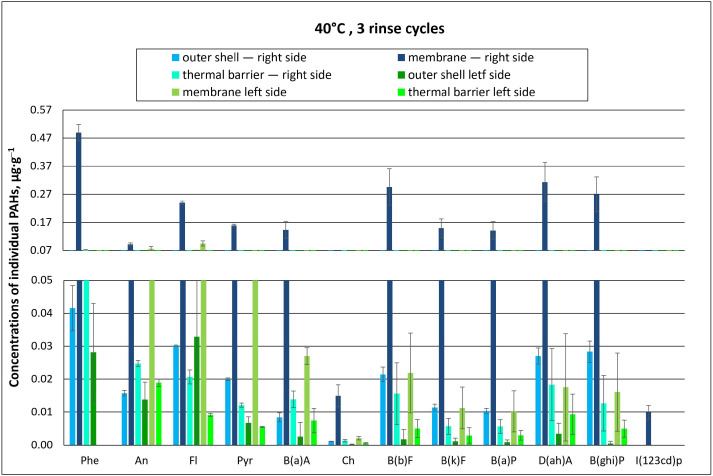
Concentrations of individual PAHs in type A material packages after exposure to chemicals and washing (40 °C, 3 rinse cycles; error bars represent standard deviations).

**Figure 10:**
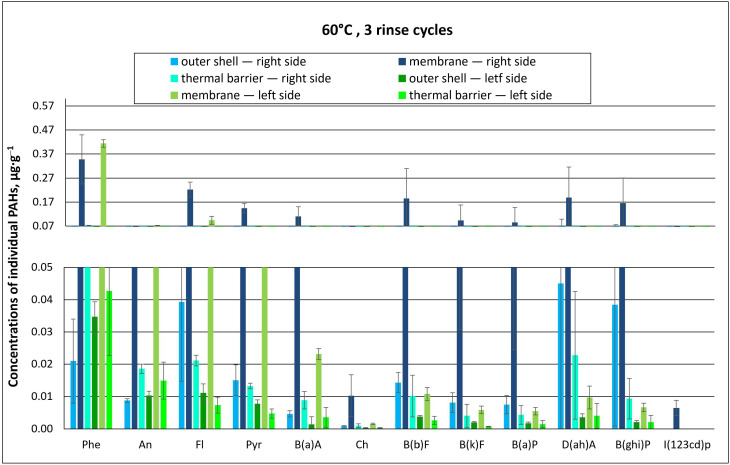
Concentrations of individual PAHs in type A material packages after exposure to chemicals and washing (60 °C, 3 rinse cycles; error bars represent standard deviations).

The concentrations of individual PAHs measured in the tested material packages are summarized in Figure 9 and Figure 10, as well as in the Supplementary Materials (Tables S3 and S4).

It was found that at both 40 °C and 60 °C, increasing the number of rinses from two to three resulted in a decreased PAH concentration in protective clothing packages. This was observed for dibenzo(a, h)anthracene (D(ah)A), characterized by a toxicity index of 5, whose concentration in the membrane of type A packages (“top side”) after washing at 40 °C with two rinse cycles was 0.41 µg∙g^−1^ (Figure 6). In comparison, after applying an additional rinse cycle, the concentration of D(ah)A decreased by about 25% to 0.31 µg∙g^−1^ (Figure 9). A similar situation was observed when washing at 60 °C. For the membrane layer, which accumulates the largest amounts of PAH contamination, the concentration of D(ah)A for type A material packages (“top side”) was 0.34 µg∙g^−1^ at two rinse cycles and approx. half as much (0.19 µg∙g^−1^) at three rinse cycles (Figure 7 and Figure 10).

For the “top side” of packages washed at 40 °C with three rinses, the highest PAH concentrations in the membrane were recorded for phenanthrene (Phe) at 0.49 µg∙g^−1^, while anthracene (An) and D(ah)A ranged from 0.092 to 0.31 µg∙g^−1^, respectively, and the concentration of benzo(a)pyrene (B(a)A) was 0.14 µg∙g^−1^ (Figure 9). For the “bottom side” of type A material packages washed at 40 °C with three rinses, the highest concentrations were also recorded in the membrane with 0.09 µg∙g^−1^ for fluoranthene (Fl).

In the case of washing the same packages at 60 °C with three rinse cycles, the concentration of individual PAHs was also the highest in the membrane for phenanthrene (Phe) at 0.35 µg∙g^−1^. The lowest concentrations of 0.0010–0.063 µg∙g^−1^ were recorded in thermal insulation samples for chrysene (Ch) and phenanthrene (Phe), respectively (Figure 10). The concentration of the PAH indicator, i.e., B(a)P, in the thermal insulation was 0.0040 µg∙g^−1^.

#### 3.2.2. Effect of the Number of Rinse Cycles on Total PAH Concentration

It was found that the use of a different number of rinse cycles in the process of washing type A material packages (two or three rinse cycles) had an effect on the obtained total PAH concentration (Figure 8). As expected, regardless of the washing temperature (40 °C or 60 °C), the higher number of rinse cycles decreased the total PAH concentration as compared to two rinse cycles. This was noted for all types of package materials (outer shell, membrane, thermal insulation), but not all cases were statistically significant.

Considering the use of a different number of rinse cycles (two or three cycles) in the process of washing package materials at 40 °C, the greatest difference in total PAH concentration was observed for the outer shell. When the packages were washed with two rinse cycles, the total PAH concentration reached 0.98 µg-g^−1^, while with three rinse cycles, this value decreased by 60%, to 0.43 µg-g^−1^ (Figure 8). In the case of the membrane, the difference between two and three rinse cycles was almost four times lower and amounted to approx. 17% (5.3 µg-g^−1^ vs. 4.4 µg-g^−1^) (Figure 8). These differences were not statistically significant (with *p*-values ranging from 0.1350 to 0.4995).

As regards different numbers of rinse cycles when washing at 60 °C, a higher total PAH concentration was noted for two rinse cycles, similarly to the washing process at 40 °C (Figure 8). The differences between the concentrations of summed PAH compounds determined in the package materials washed at 60 °C, but using different numbers of rinses, were 0.05 µg-g^−1^ for the outer shell, 0.86 µg-g^−1^ for the membrane, and 0.06 µg-g^−1^ for the thermal barrier.

The study confirmed that the number of rinse cycles influenced the total PAH concentration, mainly in the membrane material. Using three rinse cycles increased the effectiveness of the washing process for this material layer, which is important because the largest amounts of PAH compounds tend to accumulate there and are the most difficult to remove. It should be noted that the standard ISO 23616:2022 [30] which provides guidance for cleaning firefighter protective clothing, recommends three rinse cycles.

#### 3.2.3. Effect of Material Composition on Total PAH Concentration

**Figure 11:**
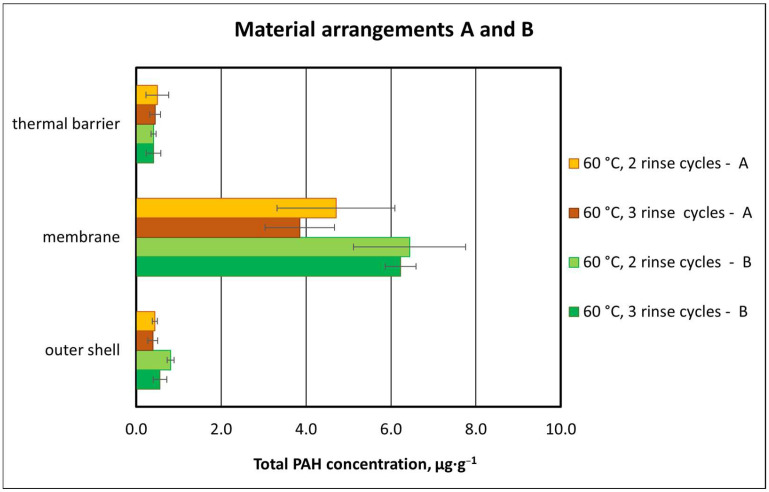
Total PAH concentrations for type A and B material packages following exposure to PAHs and washing (means plus standard deviations).

The results of the study show that, as in the case of type A packages, total PAH concentration for type B material packages did not differ considerably depending on the number of rinse cycles. The number of rinse cycles (two or three) at 60 °C was found to have little effect on the total PAH concentration in type B packages (Figure 11). In the case of the outer shell and membrane, the use of a higher number of rinse cycles (three cycles) resulted in slightly lower values for the concentration of total PAH compounds compared to the washing process with two rinse cycles (outer shell: two cycles—0.81 µg·g^−1^, three cycles—0.58 µg·g^−1^; membrane: two cycles—6.4 µg·g^−1^; three cycles—6.2 µg·g^−1^). In the case of thermal insulation, total PAH concentration was the same for both two and three cycles of washing. However, statistical analysis did not confirm the significance of such differences for any of the materials—the outer shell, membrane, or thermal insulation (with *p*-values ranging from 0.089 to 0.949).

The higher total PAH concentrations in type B packages, regardless of the number of rinses in the washing process, may have been due to their material composition, particularly due to the lower content of aramid fibers in the outer shell and membrane as compared to package A materials (Table 1, A: outer shell 98% meta-aramid, B: outer shell 58% para-aramid). Aramids are characterized by the presence of aromatic groups in the main chain and are classified as fiber-forming polyamides. As the number of aromatic groups in the structure increases, the flame, thermal, and mechanical resistance of the material improve, while its solubility decreases, which makes processing more difficult [10,31]. Aramid fibers absorb hydrophobic substances, and for this reason they are used in sorption processes and PAH extraction from solutions [32]. In addition, the materials used in type A packages were characterized by higher surface weight and thickness as compared to those in type B packages (Table 1).

The highest total PAH concentrations were recorded for the membrane in the washing variant with two rinse cycles. A slightly higher PAH concentration in the membrane layer was found in type B packages, which contained 50% polytetrafluoroethylene (PTFE) laminate, as compared to the membrane in type A packages, with 25% PTFE. Fabrics containing PTFE are characterized by high resistance to most chemicals, a wide temperature range, and a lack of water absorption. Nevertheless, the available scientific publications contain little information on the propensity of different contaminants to be sorbed by materials used in firefighter protective clothing [10]. Hence, it is difficult to say what effect the composition of the membrane material may have had on PAH absorption.

The obtained total concentrations of the tested PAH compounds were compared with the guidelines of the German Product Safety Committee [27] and the Oeko-Tex Organization [28] for maximum total PAH concentrations in materials coming in contact with the skin. The adopted 10 µg·g^−1^ limit was not exceeded for any of the materials of type A or B packages, regardless of the applied washing temperature and number of rinse cycles. The total PAH concentrations recorded after washing were 4.7–6.6 µg·g^−1^ and 7.2–7.7 µg·g^−1^ for type A and B package materials, respectively.

### 3.3. Washing Effectiveness

The washing process reduced total PAH content in the tested firefighter protective clothing packages (Table 3). The most favorable changes were observed for the washing process conducted at 60 °C with three rinse cycles:

- Package A—from 9.5 µg to 2.9 µg;
- Package B—from 9.5 µg to 4.4 µg.

Statistical analysis showed significant differences in total PAH concentrations between samples (*n* = 3) for all packages pre- and post-washing (*p* < 0.05).

The washing effectiveness of the package materials was determined based on PAH determination on the “top side” of the package and its individual layers: the outer shell, the middle membrane, and the inner thermal insulation (which are found on both the “top side” and “bottom side” of the packages). Washing effectiveness was determined from the following equation [21]:

$$Effectiveness=\frac{c_{0}-c_{1}}{c_{0}}\;\cdot 100\%$$

where

*c*_0_—Amount of PAH deposited on a package before washing;

*c*_1_—Amount of PAH found in the package materials after washing.

**Figure 12:**
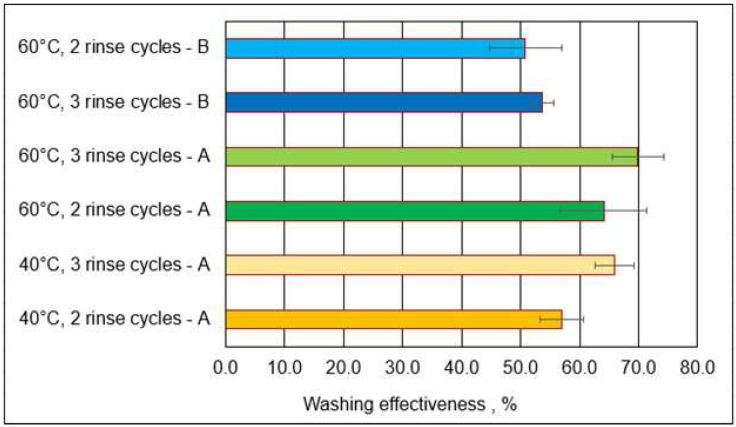
Washing effectiveness for type A and B packages following simulated exposure to chemicals and washing (means plus standard deviations).

Washing effectiveness was calculated for total PAHs, taking into account the type of package materials used in firefighter protective clothing (A or B), washing temperature, and the number of rinse cycles (Figure 12).

The results of the study show that the average washing effectiveness of type A packages varied depending on the washing temperature used and the number of rinse cycles. The washing temperature had a greater effect on the average washing effectiveness for two rinse cycles, which are used as a standard in the washing procedure (Figure 12). The use of a washing temperature of 60 °C, recommended by the protective clothing manufacturer for maintenance, had a more favorable effect on average cleaning effectiveness than a temperature of 40 °C. The difference in washing effectiveness between the washing variants at different temperatures was 7.1% (57–40 °C; 64–60 °C). However, the difference was not statistically significant (*p* = 0.27). It was observed that the number of rinse cycles (two or three) affected the average washing effectiveness of the material packages. At a given washing temperature, higher average washing effectiveness was recorded for the higher number of rinse cycles used. The difference in washing effectiveness at 40 °C between two and three washing cycles was 9.0% (57%—two cycles; 66%—three cycles).

A higher cleaning effectiveness (over 70%) was observed by Marín-Sáez et al. [33] in a proposed non-destructive sampling method for evaluating PAH exposure in firefighter personal protection equipment (PPE). The authors sampled PPE stored after a fire in sealed bags and in a dark place. They took samples immediately before or after cleaning various areas: the sleeve, chest, belly, back, gloves, and others. The samples, as in our study, were subjected to a washing process using the cleaning protocol involving an industrial washer–extractor provided by an automatic dosing of soap. The analysis showed significant PAH contamination in the glove, lower back sleeve, and knee areas due to direct exposure to flames and smoke during firefighting operations, with the highest concentrations in the outer layers. The determined washing effectiveness showed a significant reduction in PAH concentrations of about 76% [33]. Tests conducted for type B material packages, as in the case of type A packages, indicated that the introduction of an additional rinse cycle during the washing process slightly increased the effectiveness of PAH removal while maintaining the same washing temperature (Figure 12). The use of the standard two rinse cycles at a washing temperature of 60 °C led to a cleaning effectiveness of 51%. On the other hand, for three rinse cycles, about 3% higher cleaning effectiveness of 54% was achieved. However, statistical analysis did not show the differences to be significant (*p* = 0.495). Membrane A contained 25% PTFE and membrane B contained 50% PTFE. For type A material packages with membrane A with a lower content of PTFE, higher washing effectiveness (70%) was obtained in comparison to type B material packages with membrane B with twice the content of PTFE—54%. The membrane also consisted of a tightly adhered aramid layer. The authors assumed that PAH compounds accumulated in this aramid layer and the desorption of PAH compounds from the membrane was difficult due to the structure of the aramid layer (fluffy).

Similar research in this area was conducted by Hossain and Ormond [23]. They analyzed the desorption of PAHs from contaminated fabrics during the washing process, determining the partition coefficient and standard affinity between the fabric and the solution. They assessed the effect of presoaking time on the effectiveness of removing chemical contaminants from clothing. Their results indicated that with increasing presoaking time, the detergent in the bath had more time to interact with PAH molecules, which facilitated increased desorption from fabrics. Therefore, removal effectiveness significantly increased after 3 or 12 h of presoaking, to 80% for low-molecular-weight PAHs and to 51% for high-molecular-weight PAHs.

Wang et al. [34] investigated three-layer fabric structures used for firefighter clothing, similarly to the authors. Wang et al.’s research [34] concerned the effect of hydrophobicity and outer fabric structure on PAH deposition, taking into account different fabric lay angles (45°, 90°, and 180°). They observed that PAH levels detected in the middle layer (membrane) were higher than those detected in the outer fabric and inner layers, regardless of the test conditions. The polycyclic aromatic hydrocarbon content in the middle layer reached 228.9 mg/kg. In the outer and thermal barrier layers, the PAH content was 68.8 and 0.9 mg/kg, respectively. The moisture-protective membrane layer had a larger specific surface area due to its microporous structure, thus limiting the migration of PAHs. However, this resulted in the highest contamination of this layer and the greatest difficulty in removing contaminants.

In their study of laundering effectiveness for firefighter sock, underwear, and top materials, Engelsman et al. [29] recorded a 36% reduction in the concentration of total PAH compounds for socks and 9% for underwear, with a 160% increase for crop tops. The authors observed that tops made of a blend of cotton and elastane revealed a significantly higher (*p* < 0.05) post-washing total PAH concentration, suggesting cross-contamination with PAHs during the washing process. The authors did not observe an increase in total PAH concentration after the washing process in their study. An explanation for this may be that Engelsman et al. [29] used a household-type washing machine with popular laundry detergents.

## 4. Conclusions

The presented study on the effects of washing conditions on the removal of PAHs from firefighter protective clothing materials subjected to simulated contamination revealed the influence of temperature and the number of rinses on process effectiveness.

The most favorable results were obtained for the washing process conducted at 60 °C with three rinse cycles, which led to the lowest total PAH concentrations in the outer shell, membrane, and thermal insulation for both types of sample packages (A and B), differing in their material composition (0.40 µg·g^−1^ and 0.60 µg·g^−1^ of PAHs in the outer shell; 3.9 µg·g^−1^ and 6.2 µg·g^−1^ in the membrane; 0.40 µg·g^−1^ and 0.41 µg·g^−1^ in the thermal insulation, respectively). Using the higher washing temperature (60 °C) was more beneficial for average washing effectiveness than the lower temperature (40 °C) in both processes with two and three rinse cycles. The difference in washing effectiveness for type A packages between the two washing variants with two rinse cycles under different temperature conditions was 7.1% (64% at 60 °C). On the other hand, for the washing variant with three rinse cycles, it was 3.9% (70% at 60 °C).

The average washing effectiveness also varied depending on the type of materials used and their composition: it was 70% for type A packages and 54% for type B packages. The use of a third rinse cycle in addition to the standard washing procedure recommended by the garment manufacturer increased the effectiveness of PAH removal, especially from the membrane layer. This is important because that layer tends to accumulate the largest amounts of PAH compounds and is the most difficult to clean. The use of three rinse cycles is also recommended in normative documents.

The current paper presents a preliminary study. The applied method of simulating the contamination of clothing materials with harmful chemicals can be used for further research on the effects of washing parameters.

## 5. Limitations

It should be noted that samples of firefighter protective clothing materials were subjected to simulated contamination with PAH compounds, rather than actual contamination from rescue operations. Thus, the exact amount of PAH compounds deposited on the samples was known, as intended. However, this may present some difficulty when comparing test results for different methods of contaminating firefighter protective clothing materials. In the current study, this method of simulated contamination was used to avoid the influence of additional factors and accurately determine the effect of laundry process conditions on the effectiveness of removing harmful PAH compounds.

Another limitation is the small number of test samples. For testing specific conditions of the washing process (temperature, number of rinse cycles), three samples for each type of package, consisting of several material layers (outer shell, membrane, and thermal insulation), were prepared. A minimum of three samples were also taken from each individual material layer for analysis of PAH compounds. The small number of samples may not reflect all possible scenarios of clothing contamination. In some cases, the penetration of PAH solution droplets could have caused a different distribution of contaminant content across individual layers. In future research, it would be beneficial to select some specific washing process variants and increase the number of test samples.

It should be noted that it was not possible to determine PAH concentrations in the same sample before and after washing due to destructive test methods. After cutting out the sample packages before washing and taking samples for PAH analysis, the washing process with the same package sample could not be continued. The destructive nature of the testing required separate preparation of material packages for pre- and post-wash sampling.

Another limitation of the research was the lack of tests on the durability of materials after subsequent washing. The work focused on determining the content of PAH compounds in materials before and after washing. However, it would be worth checking whether the mechanical resistance of the materials to tearing and breaking remained at a satisfactory level. This could be the subject of further research.

## Figures and Tables

**Table 1:** Characterization of the materials.

Type of Clothing	Composition	Mass Per Unit Area, g∙m^−2^	Thickness, mm	Sample Weight ^(1)^, g
A	Outer shell: 98% meta-aramid, 2% antistatic fiber	218.6 ± 2.5	0.50 ± 0.01	0.35
	Moisture membrane: 50% meta-aramid, 25% para-aramid, 25% polytetrafluoroethylene (PTFE) laminate	176.5 ± 1.0	1.14 ± 0.02	0.28
	Thermal insulation: lining: 50% aramid, 50% FR viscose; felt: 85% meta-aramid, 15% para-aramid	177.7 ± 1.6	0.96 ± 0.04	0.28
B	Outer shell: 58% para-aramid, 40% polybenzimidazole (PBI), 2% antistatic fiber	200.2 ± 1.0	0.45 ± 0.01	0.32
	Moisture membrane: 25% meta-aramid, 25% para-aramid, 50% polytetrafluoroethylene (PTFE) laminate	107.9 ± 1.9	0.61 ± 0.03	0.17
	Thermal insulation: lining: 93% meta-aramid, 5% para-aramid, 2% antistatic fiber; felt: 67% meta-aramid, 33% para-aramid	167.9 ± 1.7	0.84 ± 0.02	0.27

^(1)^ Sample surface area: 0.0016 m^2^.

**Table 2:** Content of individual PAHs in 250 µL of solution applied onto material packages [23].

PAH	Abbreviation	PAH Content Dripped Onto Package, µg	Molecular Weight, g·mol^−1^	Number of Aromatic Rings	Solubility in Water, mg·L^−1^
Naphthalene	Naph	2.5	128.17	2	31.000
Acenaphthylene	Acn	2.5	152.20	3	3.800
Fluorene	Flu	0.5	166.20	3	1.900
Phenanthrene	Phe	0.25	178.23	3	1.100
Anthracene	An	0.25	178.23	3	0.045
Fluoranthene	Fl	0.5	202.25	4	0.260
Pyrene	Pyr	0.25	202.26	4	0.132
Benz(a)anthracene	B(a)A	0.25	228.29	4	0.011
Chrysene	Ch	0.25	228.29	4	0.001
Benzo(b)fluoranthene	B(b)F	0.5	252.32	5	0.001
Benzo(k)fluoranthene	B(k)F	0.25	252.32	5	0.001
Benzo(a)pyrene	B(a)P	0.25	252.32	5	0.004
Dibenzo[a,h]anthracene	D(ah)A	0.5	278.35	5	0.001
Benzo(g,h,i)perylene	B(ghi)P	0.5	276.33	6	0.0002
Indeno(1,2,3-cd)pyrene	I(123cd)p	0.25	276.33	6	0.062
Total		9.5			

**Table 3:** Total PAH content in the packages of firefighter protective clothing materials.

Type of Clothing	Washing Conditions	Package Number	Total PAH Content, µg (µg PAH per g of Package)	
			Dripped Onto the Package Before Washing	In the Package After Washing
A	40 °C, 2 rinse cycles	1	9.5	4.5
		2	9.5	3.9
		3	9.5	3.9
		Mean	9.5	4.1
		Standard deviation	0.00	0.35 ^(1)^
	40 °C, 3 rinse cycles	1	9.5	3.5
		2	9.5	2.9
		3	9.5	3.3
		Mean	9.5	3.2
		Standard deviation	0.00	0.31 ^(1)^
	60 °C, 2 rinse cycles	1	9.5	4.0
		2	9.5	2.7
		3	9.5	3.5
		Mean	9.5	3.4
		Standard deviation	0.00	0.70 ^(1)^
	60 °C, 3 rinse cycles	1	9.5	3.0
		2	9.5	3.2
		3	9.5	2.4
		Mean	9.5	2.9
		Standard deviation	0.00	0.41 ^(1)^
B	60 °C, 2 rinse cycles	1	9.5	5.3
		2	9.5	4.6
		3	9.5	4.1
		Mean	9.5	4.7
		Standard deviation	0.00	0.58 ^(1)^
	60 °C, 3 rinse cycles	1	9.5	4.4
		2	9.5	4.2
		3	9.5	4.6
		Mean	9.5	4.4
		Standard deviation	0.00	0.19 ^(1)^

^(1)^ Standard deviation means margin of error.

## Data Availability

The original contributions presented in this study are included in the article/Supplementary Materials. Further inquiries can be directed to the corresponding authors.

## Supplementary Materials

The following supporting information can be downloaded at: https://www.mdpi.com/article/10.3390/ma18174073/s1, Table S1: Drawing data to Figure 6; Table S2: Drawing data to Figure 7; Table S3: Drawing data to Figure 9; Table S4: Drawing data to Figure 10.
