# Supplementary material for: Effects of Washing Conditions on PAH Removal Effectiveness in Firefighter Protective Clothing Materials
Source: Materials (Basel). 2025 Aug 30;18(17):4073. doi: 10.3390/ma18174073 (PMC12429547; doi:10.3390/ma18174073)
Supplement: Supplementary file 1 [file materials-18-04073-s001.zip › materials-3824784-supplementary.pdf]

SUPPLEMENTARY MATERIALS

Drawing data

Table S1 Drawing data to Figure 6

No	PAH name	PAH concentrations, $\mu g \cdot g^{-1}$ (µg PAH per g of sample)					
		outer shell – right side	outer shell – left side	membrane - right side	membrane - left side	thermal barier – right side	thermal barier – left side
1	Naphthalene	0.000	0.000	0.000	0.000	0.000	0.000
2	Acenaphthylene	0.000	0.000	0.000	0.000	0.000	0.000
3	Fluorene	0.000	0.000	0.000	0.000	0.000	0.000
4	Phenanthrene	0.038	0.024	0.443	0.000	0.036	0.041
5	Anthracene	0.017	0.010	0.096	0.091	0.020	0.018
6	Fluoranthene	0.027	0.016	0.304	0.129	0.020	0.012
7	Pyrene	0.019	0.024	0.184	0.084	0.012	0.007
8	Benz(a)anthracene	0.010	0.003	0.195	0.027	0.012	0.011
9	Chrysene	0.001	0.000	0.020	0.002	0.001	0.000
10	Benzo (b)fluoranthene	0.043	0.004	0.402	0.015	0.010	0.004
11	Benzo(k)fluoranthene	0.022	0.001	0.203	0.008	0.004	0.002
12	Benzo(a)pyrene	0.019	0.001	0.180	0.007	0.004	0.002
13	Dibenzo[a,h]anthracene	0.049	0.001	0.406	0.010	0.008	0.006
14	Benzo(g,h,i)perylene	0.054	0.000	0.352	0.009	0.006	0.004
15	Indeno(1,2,3-cd)pyrene	0.000	0.000	0.011	0.000	0.000	0.000

Table S2 Drawing data to Figure 7

No	PAH name	PAH concentrations, $\mu g \cdot g^{-1}$ (µg PAH per g of sample)					
		**outer shell – right side**	**outer shell – left side**	**membrane - right side**	**membrane - left side**	**thermal barier – right side**	**thermal barier – left side**
1	Naphthalene	0.000	0.000	0.000	0.000	0.000	0.000
2	Acenaphthylene	0.000	0.000	0.000	0.000	0.000	0.000
3	Fluorene	0.000	0.000	0.000	0.000	0.000	0.000
4	Phenanthrene	0.029	0.043	0.400	0.000	0.020	0.066
5	Anthracene	0.014	0.015	0.071	0.093	0.013	0.020
6	Fluoranthene	0.060	0.018	0.257	0.153	0.013	0.020
7	Pyrene	0.022	0.011	0.170	0.094	0.008	0.012
8	Benz(a)anthracene	0.009	0.000	0.165	0.031	0.007	0.010
9	Chrysene	0.001	0.000	0.017	0.002	0.001	0.001
10	Benzo (b)fluoranthene	0.019	0.004	0.332	0.020	0.005	0.013
11	Benzo(k)fluoranthene	0.009	0.002	0.140	0.011	0.002	0.004
12	Benzo(a)pyrene	0.009	0.002	0.155	0.010	0.002	0.001
13	Dibenzo[a,h]anthracene	0.019	0.004	0.340	0.019	0.004	0.028
14	Benzo(g,h,i)perylene	0.023	0.004	0.301	0.015	0.003	0.016
15	Indeno(1,2,3-cd)pyrene	0.000	0.000	0.009	0.000	0.000	0.000

Table S3 Drawing data to Figure 9

No	PAH name	PAH concentrations, μg·g^{-1} (μg PAH per g of sample)					
		**outer shell – right side**	**outer shell – left side**	**membrane - right side**	**membrane - left side**	**thermal barier – right side**	**thermal barier – left side**
1	Naphthalene	0.000	0.000	0.000	0.000	0.000	0.000
2	Acenaphthylene	0.000	0.000	0.000	0.000	0.000	0.000
3	Fluorene	0.000	0.000	0.000	0.000	0.000	0.000
4	Phenanthrene	0.042	0.028	0.489	0.000	0.069	0.000
5	Anthracene	0.016	0.014	0.092	0.076	0.025	0.019
6	Fluoranthene	0.030	0.033	0.240	0.095	0.021	0.009
7	Pyrene	0.020	0.007	0.159	0.066	0.012	0.006
8	Benz(a)anthracene	0.008	0.003	0.143	0.027	0.014	0.007
9	Chrysene	0.001	0.000	0.015	0.002	0.001	0.001
10	Benzo (b)fluoranthene	0.021	0.002	0.296	0.022	0.016	0.005
11	Benzo(k)fluoranthene	0.011	0.001	0.149	0.011	0.006	0.003
12	Benzo(a)pyrene	0.010	0.001	0.141	0.010	0.006	0.003
13	Dibenzo[a,h]anthracene	0.027	0.003	0.313	0.018	0.018	0.009
14	Benzo(g,h,i)perylene	0.028	0.000	0.270	0.016	0.013	0.005
15	Indeno(1,2,3-cd)pyrene	0.000	0.010	0.000	0.000	0.002	0.000

Table S4 Drawing data to Figure 10

No	PAH name	PAH concentrations, $\mu g \cdot g^{-1}$ (µg PAH per g of sample)					
		outer shell – right side	outer shell – left side	membrane - right side	membrane - left side	thermal barier – right side	thermal barier – left side
1	Naphthalene	0.000	0.000	0.000	0.000	0.000	0.000
2	Acenaphthylene	0.000	0.000	0.000	0.000	0.000	0.000
3	Fluorene	0.000	0.000	0.000	0.000	0.000	0.000
4	Phenanthrene	0.021	0.035	0.348	0.414	0.063	0.043
5	Anthracene	0.009	0.010	0.052	0.066	0.019	0.015
6	Fluoranthene	0.039	0.011	0.222	0.094	0.021	0.007
7	Pyrene	0.015	0.008	0.144	0.064	0.013	0.005
8	Benz(a)anthracene	0.005	0.001	0.111	0.023	0.009	0.004
9	Chrysene	0.001	0.000	0.010	0.002	0.001	0.000
10	Benzo (b)fluoranthene	0.014	0.004	0.185	0.011	0.010	0.003
11	Benzo(k)fluoranthene	0.008	0.002	0.094	0.006	0.004	0.001
12	Benzo(a)pyrene	0.008	0.002	0.086	0.005	0.004	0.005
13	Dibenzo[a,h]anthracene	0.045	0.004	0.189	0.010	0.023	0.004
14	Benzo(g,h,i)perylene	0.038	0.002	0.165	0.007	0.009	0.002
15	Indeno(1,2,3-cd)pyrene	0.000	0.000	0.006	0.000	0.000	0.000
